# Genotypes and associated traits in *Salmonella enterica* Newport strains linked to fresh produce-associated outbreaks in the United States

**DOI:** 10.3389/fmicb.2025.1676706

**Published:** 2026-01-23

**Authors:** Michelle Qiu Carter, Diana Carychao, Lisa Gorski, Rebecca L. Lindsey, James L. Bono

**Affiliations:** 1 USDA, Agricultural Research Service, Western Regional Research Center, Produce Safety and Microbiology Research Unit, Albany, CA, United States; 2 U.S. Centers for Disease Control and Prevention, Enteric Diseases Laboratory Branch, Atlanta, GA, United States; 3 USDA, Agricultural Research Service, U.S. Meat Animal Research Center, Meat Safety and Quality Research Unit, Clay Center, NE, United States

**Keywords:** *Salmonella enterica*, Newport, comparative genomics, biofilm, adhesins, virulence genes, fimbriae, pathogenicity islands

## Abstract

*Salmonella enterica* is a leading cause of bacterial infection in humans and animals. Newport is among the most prevalent serotypes linked to fresh produce-associated salmonellosis outbreaks in the United States and among the top serotypes that cause foodborne outbreaks overall. In this study, comparative pathogenomic analyses and phenotypic assays were performed to uncover genetic and phenotypic traits contributing to pathogenicity and epidemiological prevalence of Newport. The 10 clinical strains were placed in four sequence types (ST5, ST31, ST45, and ST118) using classical MLST method and 10 SNP clusters using NCBI Pathogen Detection pipeline. Of the 10 SNP clusters, several persistent genotypes were identified, including PDS000127718 and PDS000029636, and each contained more than 4,000 matched strains and had been detected over a long period of time (> 20 years). In contrast, some SNP clusters appeared to represent transient genotypes, such as PDS000002512 that contained less than 10 matched strains and had been detected within a short period of time (< 5 years). The core virulence determinants in Newport included SPI-1 and SPI-2 encoded T3SS, SPI-4 and SPI-9 encoded T1SS, SPI-6 encoded T6SS, and many fimbriae and nonfimbrial adhesins. Among the seven SPIs detected, SPI-6 exhibited the greatest sequence divergence, including a large deletion that abolished both T6SS and Saf fimbriae simultaneously. Of the 11 fimbriae examined, Peg and Ste fimbriae genes were detected only in the lineage II strains while Stc fimbriae genes were detected only in the lineage III strains. Vast strain variation was revealed in expression of curli fimbriae, biofilm formation, and adherence to cantaloupe rind. Expression of curli fimbriae appeared to be strain-specific and was not associated with ST or lineage. Under the condition tested, curli enhanced biofilm formation significantly but tempered adherence of Newport to cantaloupe rind, implying a role of other adhesins in the initial interaction between Newport cells and the surface of cantaloupe rind. More accessory genes were identified in strains with a persistent genotype than in strains with a transient genotype, suggesting a role of accessory genes in dissemination of *S. enterica* Newport.

## 1 Introduction

Non-typhoidal *Salmonella enterica* remains one of the leading causes of bacterial foodborne illness around the world, causing an estimated 1.3 million cases each year in the United States (Scallan Walter et al., 2025). *S. enterica* infections are primarily transmitted through ingestion of contaminated food or water, and less commonly through contact with infected animals or people. Analyses of surveillance data reported to the Centers for Disease Control and Prevention (CDC) in 2017 revealed the majority of salmonellosis was transmitted through contaminated food (Beshearse et al., 2021). Poultry and eggs were common sources, while nuts and seeds, sprouts, and fruits had often been associated with multistate outbreaks (Snyder et al., 2019). Fresh produce has increasingly been recognized as a vehicle of transmission in the United States and other parts of the world. Between 2009 and 2015, there were nearly 900 documented foodborne illness outbreaks due to *Salmonella* contamination with seeded vegetables being the second most common food type implicated (Dewey-Mattia et al., 2018). In a recent study that applied an ensemble machine learning algorithm (Supervised random forest model) to determine the source attribution of human salmonellosis cases (Rose et al., 2025), chicken and vegetables were predicted to be the main sources.

*S. enterica* is an extremely diverse species containing over 2,600 serotypes, with most human illness due to approximately 100 serotypes (European Food Safety Authority, 2022; Grimont and Weill, 2007; CDC, 2025). Newport is one of a few serotypes responsible for a majority of human salmonellosis in the United States (Andino and Hanning, 2015; Crim et al., 2018; Medalla et al., 2021). In 2024, Newport was found to be the second most common serotype associated with human illness (CDC, 2025). Moreover, Newport is among the top five serotypes that cause foodborne outbreaks overall (CDC, 2018; Trees et al., 2024; Luvsansharav et al., 2020). Based on the data reported to the Foodborne Disease Outbreak Surveillance System from 1998–2013, Newport was one of the most prevalent serotypes isolated from the select commodities of fresh fruit and vegetables collected between 2002 and 2012, and was the serotype associated with most fruit and vegetable-related outbreaks in the United States (Bennett et al., 2018; Reddy et al., 2016). Of the 308 outbreaks of Newport infections reported to the National Outbreak Reporting System (NORS) from 1972–2023, nearly half of the outbreaks with confirmed food vehicles were fruits and vegetables (CDC, 2025). The implicated fresh produce has expanded from tomatoes, melons, sprouts, and cucumbers, to less common produce such as cilantro, parsley, onions, and peppers (Hanning et al., 2009; Fatica and Schneider, 2011; McCormic et al., 2022; Jenkins et al., 2023; Denich et al., 2024; Angelo et al., 2015).

Contamination of fresh produce can occur at any stage of production and from various sources, such as irrigation water, soil amendments, and animal intrusion in preharvest fields; wash water, and food contact surfaces in processing and packing areas in postharvest processing facilities. Persistence of *S. enterica* on fresh produce is impacted by both physiochemical properties of plant surfaces and the ability of *S. enterica* to attach to and colonize the plant (Truschi et al., 2024; Yaron and Romling, 2014). For example, the population of *S. enterica* on old lettuce leaves were higher than on the younger ones and these differences were associated with leaf vein, stomatal densities, leaf surface hydrophobicity, and leaf surface soluble protein concentrations (Hunter et al., 2015). In a review of melon-associated outbreaks from 1972 to 2011, more than half were associated with cantaloupe regardless of the fact that consumption of cantaloupe was consistently below watermelon throughout the survey period (Walsh et al., 2014). This association was thought to be attributed at least in part to the surface structure of cantaloupe, a netted, mesh-like pattern across the rind. The textured rind appears to be an excellent micro-environment for pathogen survival as the netting structure traps organic debris and bacteria, hindering sanitizer penetration (Solomon and Sharma, 2009; Korir et al., 2020).

Attachment is the first step in colonization of plant surfaces. Attachment efficiency varies substantially among *Salmonella* serotypes and even among strains of the same serotype. For example, Enteritidis, Typhimurium, and Senftenberg attach more efficiently to basil leaves than Arizona, Heidelberg, or Agona (Berger et al., 2009), whereas serotype Tennessee exhibits greater adherence to lettuce than Braenderup, Negev, or Newport (Patel and Sharma, 2010). These differences could be explained in part by the differences in their fimbriae and/or adhesin genes repertoire. *S. enterica* carry numerous genes encoding fimbrial and nonfimbrial adhesins. Expressions of those adhesins are often niche-dependent and regulated by environmental cues. Some adhesins contribute to mammalian or avian host colonization, while others are involved in plant attachment and colonization (Thomas et al., 2024; Yue et al., 2012; Wiedemann et al., 2014). To date, nearly 40 fimbrial gene clusters and more than 10 protein adhesins have been described in *S. enterica,* although conditions promoting expression of these fimbriae are not fully revealed (Yue et al., 2012). Among the fimbrial adhesins, curli, the long aggregative fimbriae that are commonly present in *Enterobacteriaceae*, promote surface attachment and biofilm formation in many species including *S. enterica* (Zogaj et al., 2003; Barnhart and Chapman, 2006). Considering that cells embedded in the extracellular matrix of biofilms are more resistant to disinfection treatments, biofilms of *S enterica* are likely contributing to the persistence of this pathogen and may serve as a source of contamination in food production and processing environments (Koukkidis et al., 2017; Yaron and Romling, 2014).

*S. enterica* serotype Newport consists of genetically diverse strains. Multi-Locus Sequence Typing (MLST) based populations structure analysis revealed three main lineages, with lineage I more prevalent among humans in Europe than in North American, lineage II preferentially associated with nonhuman mammals or reptiles, and lineage III linked to humans in North American (Sangal et al., 2010). Moreover, several studies suggested that each lineage might have evolved independently and displayed a geographic structure, with the lineage specific traits mainly attributed to the repertoire of prophages, pathogenicity islands, and fimbrial gene clusters (Zheng et al., 2017; Cao et al., 2013). Although factors and mechanisms underlying the clinical and epidemiological prevalence of *S. enterica* Newport are not fully understood, certain genomic and phenotypic traits have been suggested to play a role. In this study, we assembled a set of clinical Newport strains that were linked to produce-associated outbreaks or sporadic infections in the United States to identify genomic and phenotypic traits potentially contributing to produce-associated outbreaks of Newport infections.

## 2 Materials and methods

### 2.1 Bacterial strains, reagents, and growth media

*S. enterica* Newport strains used in this study are listed in Table 1. The strains were routinely maintained and cultured in Luria-Bertani half-salt (5 g NaCl/liter) (LB).

**Table 1:** Genomic characteristics *of S. enterica* Newport clinical strains used in this study.

Strains /WGS IDs	Isolation year	^a^Transmission vehicles	BioSample	^b^Replicon sizes (bp) / # of CDSs	^c^Sequence Type /lineage	^d^SNP cluster (# of matched isolates)	^e^Genotypes	^f^References
SL254	2000	Beef	SAMN02604062	**4,827,641/4,593;** **176,473/196;** **3,605/2**	ST45/II	PDS000002504.632 (1477)	Persistent	Fricke et al. (2011)
2010K-0904	2010	Tomatoes	SAMN09761699	48 contigs/4,686	ST45/II	PDS000002512.24 (8)	Transient	EDLB_CDC
2012AM-0809	2012	Tomatoes*	SAMN41071397	**4,754,345/4,482;** **5,211/6**	ST45/II	PDS000091336.3 (7)	Transient	*This study
2010K-1120	2010	Alfalfa sprouts	SAMN04859028	28 contigs/4,498	ST118/III	PDS000004424.42 (89)	Intermediate	EDLB_CDC
2012K-1235	2012	Cantaloupe	SAMN41071398	**4,778,801/4,511;** **3,338/4**	ST118/III	PDS000004406.483 (804)	Persistent	This study
2014K-0468	2014	Chia seed powder	SAMN03177805	30 contigs/4,535	ST118/III	PDS000002569.25 (65)	Intermediate	EDLB_CDC
2014K-0684	2014	Cucumbers	SAMN03067523	**4,883,718/4,662**	ST118/III	PDS000127718.361 (4904)	Persistent	This study
2017K-1226 / PNUSAS028767	2017	Pre-cut watermelon	SAMN08134360	88 contigs/4,497	ST118/III	PDS000029581.120 (191)	Intermediate	EDLB_CDC
2013K-0316	2013	Romaine lettuce*	SAMN22365112	30 contigs/4,631/	ST5/III	PDS000042896.6 (7)	Transient	EDLB_CDC
2017K-0725 / PNUSAS021698	2017	Watermelon*	SAMN07561515	43 contigs/4,565	ST5/III	PDS000029636.1002 (4292)	Persistent	EDLB_CDC
2018K-0489 / PNUSAS037512	2018	Sprouts	SAMN08964048	46 contigs/4,737	ST31/II	PDS000019695.19 (52)	Intermediate	EDLB_CDC

^a^Transmission vehicles that are not confirmed are labelled with a *; ^b^Genome size is only reported for strains having completed genomes (reference strain SL254 and the three strains sequenced in this study, shown in bold). For draft genomes, the number of contigs are reported; ^c^The ST was determined using MLST configuration for *Salmonella enterica* at Center for Genomic Epidemiology and the lineage was assigned according to a previous study (Sangal et al., 2010); ^d^The SNP cluster was identified in Pathogen Detection using *Salmonella enterica* database. The number in parentheses represents the number of matched isolated within each SNP cluster at the time of examination (February 2025); ^e^Defined arbitrarily based on the number of matched isolates. An SNP cluster that had less than 50 matched isolates at the time of analysis was a “transient” genotype while an SNP cluster that had more than 500 match isolates at the time of analysis was a “persistent” genotype; ^f^EDLB-CDC refers to Enteric Disease Laboratory Branch (EDLB) at the Centers for Disease Control and Prevention (CDC). *Strain 2012AM-0809 was previously sequenced (Campbell et al., 2018), however, only the complete genome sequence determined in this study was used.

### 2.2 Genome sequencing, annotation, and genotyping

Genomes of *S. enterica* Newport strains 2012AM-0809, 2012K-1235, and 2014K-0684 were sequenced on a PacBio Sequel IIe system as described previously (Carter et al., 2023). Briefly, bacterial DNA was extracted from exponential phase cultures grown in LB broth using Qiagen Genomic-tip 100/G columns (Valencia, CA). Purified genomic DNA (10 μg) was sheared to a 30 Kb target fragment length using g-TUBEs (Covaris, Woburn, MA) and concentrated with 0.45x volume AMPure PB beads (Pacific Biosciences). Five μg of sheared DNA was used to make PacBio sequencing libraries using the SMRTbell Prep Kit 3.0 according to the manufacturer’s protocol and barcoded using the SMRTbell barcoded adapter plated 3.0. The Sequel II binding kit 3.2 and Sequel II sequencing plate 2.0 were used to run the library with the application HiFi reads and a 30-h movie time with a 6-h pre-extension. PacBio reads were assembled using Microbial Genome Analysis in SMRT analysis v 10.1 and contigs imported into Geneious Prime® (Dotmatics). The overlapping sequence on the ends of the contigs were removed from the 5′ and 3′ ends to generate circularized chromosomes and plasmids. The closed chromosome and plasmids were manually polished by mapping Illumina and PacBio reads to the chromosome and known plasmids using Geneious mapper. Unused reads were *de novo* assembled using the Geneious assembler for small plasmid identification. All genomes and plasmids were annotated with the NCBI Prokaryotic Genome Annotation Pipeline (Tatusova et al., 2016). The GenBank accession numbers are listed in Table 1. MLST analyses were conducted using MLST 2.0 service at the Center for Genomic Epidemiology with the *Salmonella enterica* configuration. The SNP cluster was identified using NCBI Pathogen Detection for *Salmonella enterica*.

### 2.3 Analyses of *Salmonella* pathogenicity islands (SPIs), prophages, virulence genes, and adherence genes

A total of 17 SPIs described in *Salmonella* (Kombade and Kaur, 2021) were used as queries to search against a custom database containing all genomes examined in this study by performing BLASTn in Geneious Prime®. When a complete SPI was not detected, each CDS encoded by the query SPI was used to search the genome of the testing strain by BLASTP to reveal if any homologs were present in the genome of the testing strain. The complete genome sequences were submitted to PHASTEST (Wishart et al., 2023) for identification of prophage and prophage-like elements. The common and strain specific genes were revealed by comparative genomic analyses using Edgar 3.0 with the default setting (Blom et al., 2016; Dieckmann et al., 2021). To identify virulence and adherence genes in *S. enterica* Newport, the known *S. enterica* virulence and adherence genes in The Virulence Factor DataBase (VFDB) were retrieved and used as queries to search each against a custom database containing all genomes described in this study by performing BLASTn in Geneious Prime® with a threshold of 80% for gene coverage and 70% for sequence identity.

### 2.4 Swimming motility, production of curli fimbriae, and biofilm formation

Swimming motility was examined as described previously (Carter et al., 2024). Briefly, single colonies of each *S. enterica* Newport strain were point-inoculated on soft LB agar plates (0.3%) using sterile toothpicks. The plates were incubated at 37 °C or 28 °C for 24 h prior to observing the motility. Curli fimbriae were detected by growing each strain at 28 °C for 48 h on the Congo Red indicator (CRI) plates, consisting of LB agar plates without sodium chloride (LBNS) and supplemented with 40 μg/mL of Congo Red dye and 10 μg/mL of Coomassie Brilliant Blue, as described previously (Carter et al., 2011). Curli-producing strains were indicated by red colonies whereas curli-deficient strains were indicated by white colonies on CRI plates. Biofilm assays were carried out as described previously (Carter et al., 2016; Carter et al., 2018). Briefly, 1 ml of LBNS broth inoculated with 1×10^6^ cells/mL was aliquoted into a borosilicate glass tube and then incubated statically at 28 °C for 48. At the end of each incubation, the planktonic cells were removed carefully, and the tubes were rinsed twice with 1 ml sterile distilled water and then stained with 1 ml 0.1% crystal violet at room temperature for 30 min. The dye was then removed gently, and the tubes were washed twice with sterile distilled water. The crystal violet bound to the glass tube was solubilized in 0.5 mL of 33% acetic acid and the absorbance was determined at 570 nm using a BioTek Synergy HT microplate reader (Agilent, Santa Clara, CA). Tubes with uninoculated media served as negative controls. Each data set was the average of results from at least three biological replicates. The differences in attached biomass, represented by the absorbance at 570 nm, among the strains were assessed by the adjusted *p*-value of the Tukey’s multiple comparisons test after a One-way ANOVA test (*p* ≤ 0.05) in Prism 10 (Version 10.4.1). An unpaired t test was used when the difference between the two groups was revealed.

### 2.5 Attachment assay

Single colonies of each *S. enterica* Newport strain grown on LBNS agar plates at 28 °C for 2 days were used to prepare bacterial inoculums. *S. enterica* Newport cells were collected by a cotton swab and resuspended in potassium phosphate buffer (10 mM, pH 7.0) (KP buffer), followed by dilution of each cell suspension in KP buffer to a concentration of 0.01 OD_600_ (around 10^6^ cells/ml). The actual concentration of each bacterial inoculum was determined by plate counts. Cantaloupes were purchased from a local retail store. On the day of the experiment, cantaloupe rind was cut into 2 × 2 × 0.5 cm pieces, rinsed in sterile water once, followed by drip dry. Each piece was placed in an open petri dish in a Biosafety cabinet and rested for 30 min prior to inoculation. *S. enterica* Newport cells were inoculated by spotting 20 × 5 μL bacterial cell suspension evenly across the outface surface area for a total of 100 μL. The control pieces were inoculated with the same volume of KP buffer (100 μL). The inoculated pieces were placed in covered petri dishes and incubated at 25 °C for 1 h. At the end of the incubation, each inoculated piece was transferred into a 50 mL conical tube containing 10 mL KP buffer. The tube was inverted gently five times to wash off unattached cells. Each washed piece was transferred to a new tube containing 10 mL KP buffer. The *S. enterica* Newport cells attached to the cantaloupe rind were released into 10 mL KP buffer by vortexing the tube on a Vortex (Scientific Industries Vortex Genie 2 with a 3-inch platform) at 3200 RPM for 1 min. The released cells were quantified by plate counting on MacConkey agar supplemented with 30 mM maltose. At least four biological replicates and two technical replicates were tested for each strain. The differences in the populations of attached cells among the strains were assessed by the adjusted *p*-value of the Tukey’s multiple comparisons test after a One-way ANOVA test (*p* ≤ 0.05) in Prism 10 (Version 10.4.1). An unpaired t test was used when the difference between the two groups was revealed.

## 3 Results

### 3.1 Genotypes *S. enterica* Newport clinical strains

Genomic characteristics of each Newport strain including genome size, sequence type (ST), lineage, SNP cluster, and genotype were shown in Table 1. The genome sequences of seven Newport strains were downloaded from GenBank. For the three strains that had no genome sequences available at the time the study was conducted, complete genomes were sequenced, annotated, and deposited in GenBank. The Newport strain SL254 was used as a reference for comparative genomic analyses. Four STs were detected, including ST5, ST31, ST45, and ST118, using the classical seven loci-based MLST method (Larsen et al., 2012). ST118 appeared to be dominant as five strains were ST118, followed by ST45 and ST5 (two strains for each ST) (Table 1). Lineage of each strain was assigned as described previously (Sangal et al., 2010). ST5 and ST118 are within lineage III, and ST31 and ST45 are within lineage II. The reference strain SL254 belongs to ST45 thus it is a lineage II strain.

The relatedness of each Newport strain to other *S. enterica* Newport strains described in public databases was assessed by examining the related SNP clusters using NCBI Pathogen Detection pipeline. Each of the 10 Newport strains was placed in a unique SNP cluster that differed in the number of matched isolates, ranging from seven (e.g., strains 2012AM-0809 and 2013K-0316) to 4,904 (strain 2014K-0684) at the time of analysis (Table 1). Based on the number of the matched isolates, each SNP cluster was defined arbitrarily to a persistent, transient, or intermediate genotype (Table 1). A SNP cluster that had less than 50 matched isolates at the time of analysis was a “transient” genotype while an SNP cluster that had more than 500 match isolates at the time of analysis was a “persistent” genotype. For the three ST45 strains, the SNP cluster PDS000002504.632 that contained 1,477 matched isolates including SL254 represented a persistent genotype while SNP clusters PDS000002512.24 (eight matched strains including 2010K-0904) and PDS000091336.3 (seven matched strains including 2012AM-0809) were transient genotypes (Table 1). For PDS000002512.24, four of the seven matched strains (2010K-0904, 2010K-0905, AM42932, and AM43629) were clinical strains isolated in 2010 in the United States. For PDS000091336.3, four strains (2012AM-0809, 2012AM-0810, AM51387, and M51389) were isolated in 2012 and two (PNUSAS276373 and PNUSA278884) were isolated in 2022. The most notable differences between the 2012 and 2022 isolates were the acquisition of antibiotic resistance genes in the 2022 isolates, including genes *tetA*, *sul2*, *aadA* (partial), *aac(6),* and *dfrA14*.

**Figure 1:**
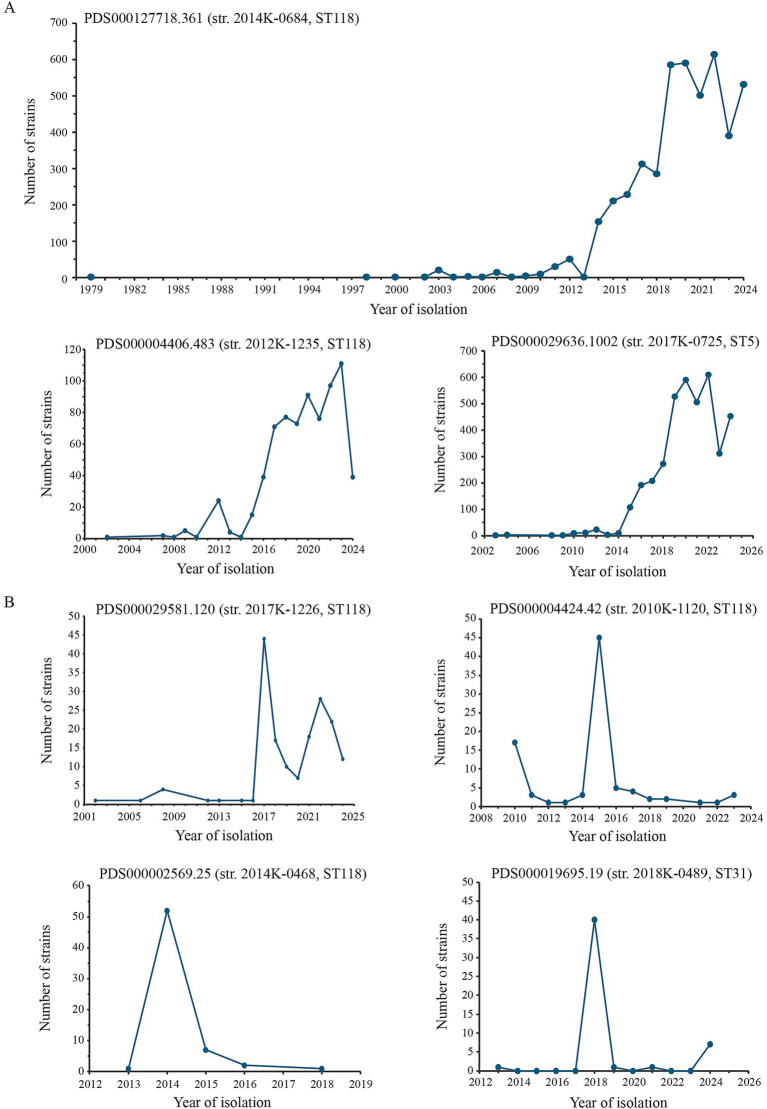
SNP clusters representing persistent and intermediate genotypes of *S. enterica* Newport. SNP clusters representing persistent **(A)** or intermediate **(B)** genotypes are presented. The SNP clusters representing transient genotypes were not graphed due to the limited numbers of matched isolates. Metadata including the collection time, sources of isolation, and geographic location of isolation for all matched strains within each SNP cluster were retrieved from the NCBI pathogen detection website. The number of matched strains within each SNP cluster are presented in Table 1. Strains collected in 2025 are not included in the figure.

For the SNP clusters containing ST118 strains, PDS000004406.483 (804 matched strains including 2012K-1235) and PDS000127718.361 (4,904 matched strains including 2014K-0684) represented persistent genotypes while the rest were intermediate genotypes (Table 1). Among the 4,904 matched strains within the SNP cluster PDS000127718.361, more than 4,500 were clinical isolates. Although the earliest collection time was traced to 1979, the frequency of isolation remained very low until 2014, when it started increasing and reached a peak in 2022 (Figure 1A). About 400 matched strains within this cluster were isolated from various environmental samples and food samples, including lettuce, cilantro, cabbage, and cucumbers, implying its environmental prevalence. Of the 804 matched strains within the SNP cluster PDS000004406.483, the majority (92.3%) were clinical isolates. Other isolation sources included animals (1.5%), environmental samples (4.5%), and food samples that were mainly represented by cantaloupe and green onion (1.6%). Most strains within this cluster were collected in the United States from 2002 to 2024 with a peak of isolation in 2023 (Figure 1A). Among the three intermediate genotypes of the ST118 strains, the SNP cluster PDS000029581.120 contained 191 matched strains including 2017K-1226, and most strains within this SNP cluster were isolated in the United States from 2002–2024 and with a peak of isolation in 2017 (Figure 1B). Similarly, most of the matched isolates within this cluster (94.2%) were clinical, and about 4.7% of the matched strains were food isolates, mainly from cucumbers. The SNP cluster PDS000004424.42 contained 89 matched strains including 2010K-1120. Unlike the three clusters discussed above, most strains within this cluster were isolated from food samples (72.3%), including alfalfa seeds in 2010, sprouts in 2011, and leafy greens in 2015 (Figure 1B). The most recently detected strains within this cluster were clinical strains isolated in the United States in 2023. The SNP cluster PDS000002569.25 contained 65 matched strains including 2014K-0468. Most of the strains within this cluster were isolated in 2014 (82.5%), and the most recent detection time was in 2018 (Figure 1B). Of the 65 matched strains, 34 were isolated from contaminated food, including chia seed powder, in the United States and Canada.

The two ST5 strains were placed in the SNP clusters PDS000042896.6 that contained seven matched isolates including 2013K-0316 and PDS000029636.1002 that contained 4,292 matched isolates including 2017K-0725 (Table 1). Of the seven PDS000042896 strains, six were clinical strains and one was an environmental strain isolated from canal water (strain FDA474828). The collection time spanned from 2009 (three) to 2024 (three). Of the 4,292 PDS000029636 strains, more than 4,000 strains were clinical, and over 100 strains were collected from diverse environmental samples including the drag swabs at cantaloupe fields. Although strains within this cluster were detected as early as 2003, the number of detected isolates did not start to increase until 2015, and reached a peak in 2022 (Figure 1A). At the time of analyses (February 2025), there were six clinical strains isolated in 2025. Therefore, SNP cluster PDS000042896 is likely a transient genotype while SNP PDS000029636 is likely a persistent genotype.

The ST31 strain 2018K-0489 was placed in the SNP cluster PDS000019695.19 that contained 52 matched strains at the time of analysis (Table 1). Most strains (> 40) were clinical and isolated in 2018 (Figure 1B). The earliest isolation time documented in Pathogen Detection was 2013 and the latest isolation time was 2021 in the United States and 2024 in Australia.

### 3.2 Comparative genomic analyses of ST45 and ST118 strains

**Figure 2:**
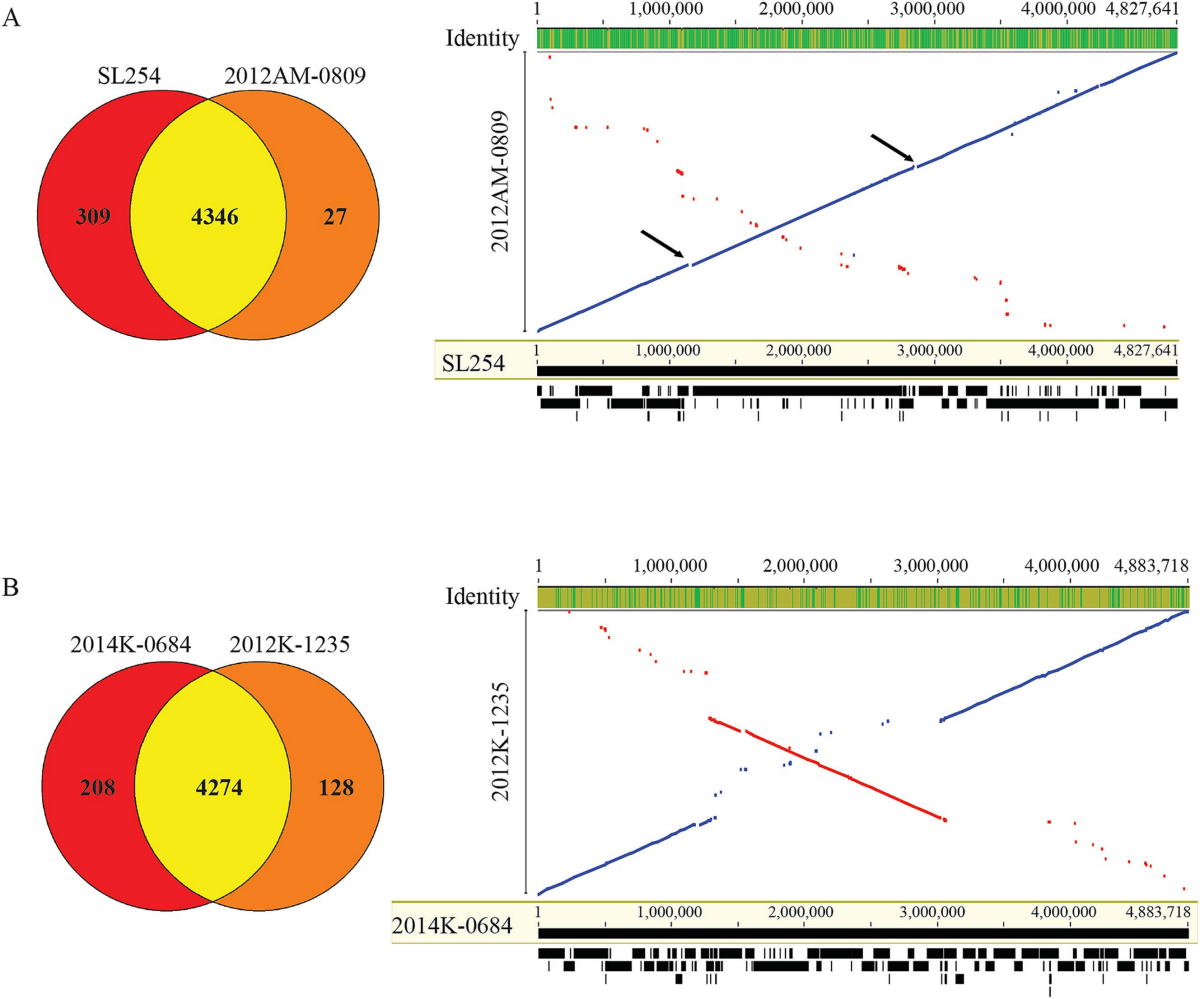
Comparative genomic analyses of ST45 and ST118 strains. **(A)** Venn diagram of shared and strain specific CDSs between the two ST45 genomes (left panel) and LASTZ alignment of the two chromosomes (right panel). Arrows indicate the large deletions detected in strain 2012AM-0809. **(B)** Venn diagram of shared and strain specific CDSs between the two ST118 genomes (left panel) and LASTZ alignment of the two chromosomes (right panel). Numbers of shared and strain specific orthologs were calculated in EDGAR 3.2 using default parameters. The numbers in LASTZ alignments indicate the aligned base pair position and the black blocks and lines at the bottom represent the 162 target DNA sequences used in the pairwise alignment of two ST45 strains **(A)** and 207 target DNA sequences used in the pairwise alignment of two ST118 strains **(B)**. The alignment was performed in Geneious Prime®2025.1.2 using LASTZ (Version 1.04.15) with the following parameters: Step Length: 20; Seed Pattern: 14 of 22; and HSP Threshold Score (upper limit): 3,000.

To gain insight into genomic traits associated with transient and persistent SNP clusters, comparative genomics were carried out for two ST5 and two ST118 strains that had a complete genome sequence (Table 1). Strain SL254 with a persistent SNP cluster had a much larger genome (5.01 Mb) than strain 2012AM-0809 with a transient SNP cluster (4.76 Mb). Strain SL254 carried more strain specific genes than strain 2012AM-0809 (Figure 2A). Of the 309 SL254 genes that had no homologs in strain 2012AM-0809, 177 genes were located on the large MDR plasmid pSN254 that confers resistances to streptomycin, gentamicin, sulfadiazine, chloramphenicol, tetracycline, and cefoxitin. Other variable genomic regions were mainly located within prophage genomes, including 56, 15, and 36 genes on the genome of the prophage 2 (Chromosomal positions: 1,135,821–1,177,764), prophage 5 (Chromosomal positions: 2,846,421–2,878,144), and prophage 6 (Chromosomal positions, 4,231,795–4,262,748), respectively (Table 2). Both variable regions on the genomes of prophage 2 (41.9 Kb) and prophage 5 (31.7 Kb) were completely missing in strain 2012AM-0809, while the variable region on the genome of prophage 6 in strain SL254 (31.0 Kb) was replaced with a divergent DNA fragment in strain 2012AM-0809 (Chromosomal positions: 595,876–564,959), exhibiting 51.8% sequence identity with the corresponding region in strain SL254. Of the 27 genes in strain 2012AM-0809 that had no homologs in strain SL254, five genes were located on the small plasmid p2012AM-0809-1, eight genes were located on the 31-Kb variable genomic region, while others were scattered throughout the chromosome.

**Table 2:** Chromosomal locations of prophages in *S. enterica* Newport strains.

^a^Prophages	^b^SL254	^b^2012AM-0809	^b^2012K-1235	^b^2014K-0684
1	47.1 Kb/ 1,057,317–1,104,492	52.4 Kb*/ 1,160,716–1,213,166	61.3 Kb*/ 1,143,673–1,205,062	29.1 Kb*/ 683,887–713,082
2	47.3 Kb/ 1,131,218–1,178,564	53.7 Kb/ 1,236,815–1,290,522	42.7 Kb*/ 1,218,745–1,261,450	64.9 Kb/ 1,172,504–1,237,492
3	22.4 Kb*/ 1,958,335–1,980,816	25.8 Kb/ 2,041,753–2,067,608	57.2 Kb/ 1,923,134–1,980,401	16.9 Kb*/ 1,284,760–1,301,676
4	66.5 Kb/ 2,715,750–2,782,335	47.1 Kb/ 2,872,772–2,919,875	47.1 Kb/ 2,919,670–2,966,859	34.8 Kb/ 1,302,089–1,336,956
5	48.6 Kb/ 2,841,212–2,889,828	18.1 Kb/ 4,375,244–4,393,396	18.1 Kb/ 4,424,992–4,443,143	49.5 Kb/ 1,515,557–1,565,110
6	32.3 Kb/ 4,230,384–4,262,735	44.3 Kb/ 4,530,048–4,574,392	NA	63.9 Kb/ 2,323,779–2,387,756
7	18.1 Kb/ 4,411,352–4,429,504	NA	NA	55.5 Kb/ 3,014,337–3,069,904
8	NA	NA	NA	12.6 Kb*/ 4,245,364–4,258,004
9	NA	NA	NA	17.4 Kb/ 4,530,458–4,547,883

^a^Prophages were identified using PHASTEST as described previously (Wishart et al., 2023). ^b^GenBank accession numbers are listed in Table 1. Data are presented in a format of “prophage genome size (Kb)/chromosomal positions.” Both questionable and incomplete prophages are marked by a “*.”NA: not applicable.

Unlike the two ST45 strains, both ST118 strains with a complete genome were from a persistent SNP cluster (Table 1). Of the 208 2014K-0684 genes that had no homologs in strain 2012K-1235 (Figure 2B), 172 were in the genomes of prophages 1–9 (Table 2), while others were scattered throughout the chromosome. Besides phages-related genes, other functions encoded by the strain specific genes in strain 2014K-0684 included transcriptional regulation, virulence, transport, and detoxification (Supplementary Table 1). Of the 128 genes present in strain 2012K-1235 but had no homologs in strain 2014K-0684 (Figure 2B), 55 were in the prophage genomes while the others (73 genes) were distributed across the chromosome. Similarly, besides the functions related to mobile genetic elements (MGEs) including prophages, the other main functions encoded by the 2012K-1235 strain specific genes included transcriptional regulation, virulence, transport, and carbon metabolism (Supplementary Table 1).

### 3.3 Comparative analyses of SPIs

Among the 17 *S. enterica* pathogenicity islands (SPIs) reported, homologs of seven (SPI-1, SPI-2, SPI-3, SPI-4, SPI-5, SPI-6, and SPI-9) were identified in the Newport strains examined in this study.

**Figure 3:**
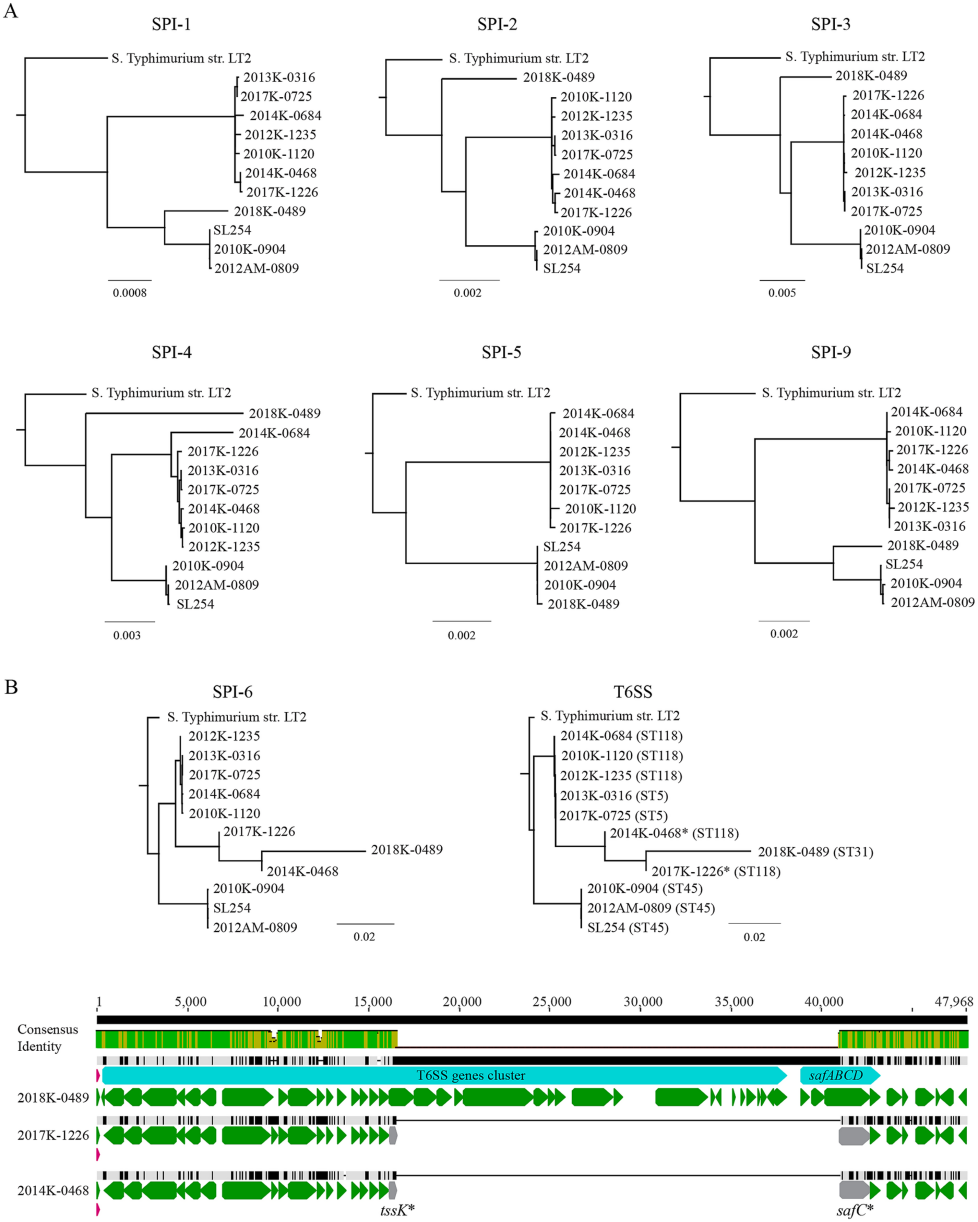
Comparative analyses of SPIs in *S. enterica* Newport strains. **(A)** Consensus trees of SPI-1, SPI-2, SPI-3, SPI-4, SPI-5, and SPI-9. **(B)** Consensus trees of SPI-6 and T6SS and the large deletion detected in the ST118 strains 2017K-1226 and 2014K-0468. Genes marked with a * represent genes carrying mutations. Sequences were aligned using Clustal Omega (1.2.3) in Geneious Prime® (2025.0.3), and a consensus tree was constructed using Jukes-Cantor method for genetic distance model and Neighbor-Joining method tree build. The tree was resampled using Bootstrap method with 10,000 replicates. Green arrows represent the annotated genes, grey arrows represent truncated genes, and pink arrows represent tRNA genes. The identity of the consensus sequence is color-coded (Green: 100% identity; Brown: at least 30% and under 100% identity; and Red: below 30% identity).

A complete SPI-1 (~ 40-Kb) was identified in all Newport strains examined, located between the genes *flhA* and *mutS*, like the SPI-1 reported in other serotypes. Both type three secretion system (T3SS) genes and the genes related to iron uptake (*sitABCD*) were detected. Phylogenetic analyses grouped the SPI-1 s in the lineage III strains (ST5 and ST118) together, while the SPI-1 s in the lineage II strains (ST31 and ST45) were placed in a separate cluster (Figure 3A, SPI-1), implying a congruent evolution of SPI-1 in *S. enterica* Newport. Like SPI-1, a complete SPI-2 (nearly 40-Kb) was detected in all Newport strains examined and located adjacent to tRNA gene *valV*. Besides the T3SS genes, the SPI-2 carried the tetrathionate reductase genes (*ttrBCA*) and the genes encoding a two-component system (TtrR-TtrS) that regulates expression of *ttrBCA*. The consensus tree of the SPI-2 exhibited a similar topograph as the SPI-1, except that the SPI-2 in strain 2018K-0489 (ST31) was placed in a lineage that was separated from all other Newport strains (Figure 3A, SPI-2). The SPI-3 in *S. enterica* Typhimurium is a 17-Kb genomic island inserted downstream of tRNA gene *selC*. A smaller SPI-3 (~13 Kb) was identified in all Newport strains and also located next to the tRNA gene *selC*. The SPI-3 in Newport strains displayed >90% sequence similarity with the SPI-3 in Typhimurium strain LT2, carrying the gene encoding autotransporter protein MisL and the genes (*mgtBC*) that are essential for the survival of the pathogen in macrophages (Blanc-Potard and Groisman, 1997). However, four genes located between the genes *selC* and *misL* in Typhimurium strain LT2 were missing in all Newport strains examined. The consensus tree of SPI-3 displayed a similar topograph as the SPI-2 (Figure 3A, SPI-3). The SPI-4 in Typhimurium strain LT2 is 23 Kb, carrying genes related to biogenesis of Type 1 secretion system (T1SS) and a large protein with repeated Ig domains (SsiE) that is secreted by the T1SS. A highly conserved SPI-4 was identified in all Newport strains (~ 93% Identity over 23.4 Kb). The consensus tree of SPI-4 displayed a similar topograph with the SPI-2. SPI-5 is a small genomic island (~ 6.8 Kb) encoding additional T3SS effector proteins that are translocated by either SPI-1 encoded T3SS (SopB) or SPI-2 encoded T3SS (PipB). A highly conserved SPI-5 was detected in all Newport strains (~ 98% Identity over 6.8 Kb). The consensus tree of SPI-5 displayed a similar topograph as the SPI-1. Like SPI-4, the SPI-9 carries genes related to biogenesis of T1SS and a gene encoding a large protein adhesin with repeated Ig domains (BapA). A highly conserved SPI-9 was detected in all Newport strains (~ 96% Identity over 16 Kb). The consensus tree of SPI-9 exhibited a similar topograph as that of SPI-1 (Figure 3A, SPI-9).

The SPI-6 is a large genomic island that carries genes encoding type six secretion system (T6SS) and genes related to biogenesis of *Salmonella* atypical fimbriae (Saf) (*safABCD*). In *S. enterica* Typhimurium strain LT2, SPI-6 is about 47 Kb and inserted next to the tRNA gene *aspV* (Folkesson et al., 2002). In Newport strains, a SPI-6 was detected at the same chromosomal location as the SPI-6 in Typhimurium strain LT2, but the size of SPI-6 varied from 23 Kb to 49 Kb. In most Newport strains, the key T6SS genes were present and located on a 34-Kb DNA fragment that was adjacent to the tRNA gene *aspV*. However, in ST118 strains 2014K-0468 and 2017K-1226, a much smaller SPI-6 was revealed, which resulted from the deletion of a 24-Kb DNA fragment spanning from gene *tssk* to gene *safC* (Figure 3B). Furthermore, unlike any of the SPIs discussed above, the SPI-6 in ST31 strain 2018K-0489 exhibited higher sequence similarity with the SPI-6 s in the lineage III strains than with SPI-6 s in other lineage II strains (Figure 3B, SPI-6). A similar trend was observed when only the T6SS genes were used for the analysis (Figure 3B, T6SS).

### 3.4 Virulence genes in *S. enterica* Newport

Among the 30 genes encoding T3SS effector proteins in *S. enterica*, homologs of 23 genes were detected in all Newport strains examined (Supplementary Table 2). Mutations were common in gene *avrA* since an amber mutation was present in eight of 10 strains examined. Mutations were detected in gene *sopA* of both ST5 strains and three ST118 strains. Additionally, mutation in gene *sspH2* was detected in strain 2010K-1120. In contrast, among the remaining seven T3SS effector genes, homologs of five (*sopE*, *gogB*, *sseI*, *sseK1*, and *sspH1*) were detected in a subset of the 10 strains examined. No homologs of *spvC*, encoding the T3SS effector phosphothreonine lyase SpvC, or *spvD*, encoding the T3SS effector cysteine hydrolase SpvD, were identified in any of the Newport strains examined.

Among the 15 other virulence factors reported in *S. enterica* (Supplementary Table 2), a distant homolog of *rck* (gene_synonym: *srgB*), *pagC,* was identified in all Newport strains. The *rck* gene in Typhimurium strain LT2 encodes an outer membrane protein that functions as an invasin, mediating bacterial entry into host cells and also conferring resistance to complement-mediated killing (Koczerka et al., 2021). PagC exhibited 52% identity with the Rck protein. No homologs of genes encoding exotoxin SpvB, CdtB, PltA, or PltB were identified in any of the Newport strains examined. Similarly, no homologs of the genes encoding any of the Vi antigens were identified in any of the Newport strains examined.

### 3.5 Adherence genes in *S. enterica* Newport

Among the ten protein adhesins genes examined, a homolog of each was identified in all strains except that in strain 2013K-0316, two copies of *misL* (LIX38_004722 and LIX38_001929), encoding an intestinal colonization autotransporter adhesin, were identified (Supplementary Table 3). Mutations in *bigA* appeared to be common in Newport considering that large deletions and/or point mutations were detected in six out of 11 strains examined, including three ST45 strains, two ST5 strains, and one ST118 strain. Other mutations included a large insertion in *ratA* of strain 2014K-0468, and a point mutation in *sadA* of strain 2010K-1120 and in *sinH* of strain 2014K-0684.

**Figure 4:**
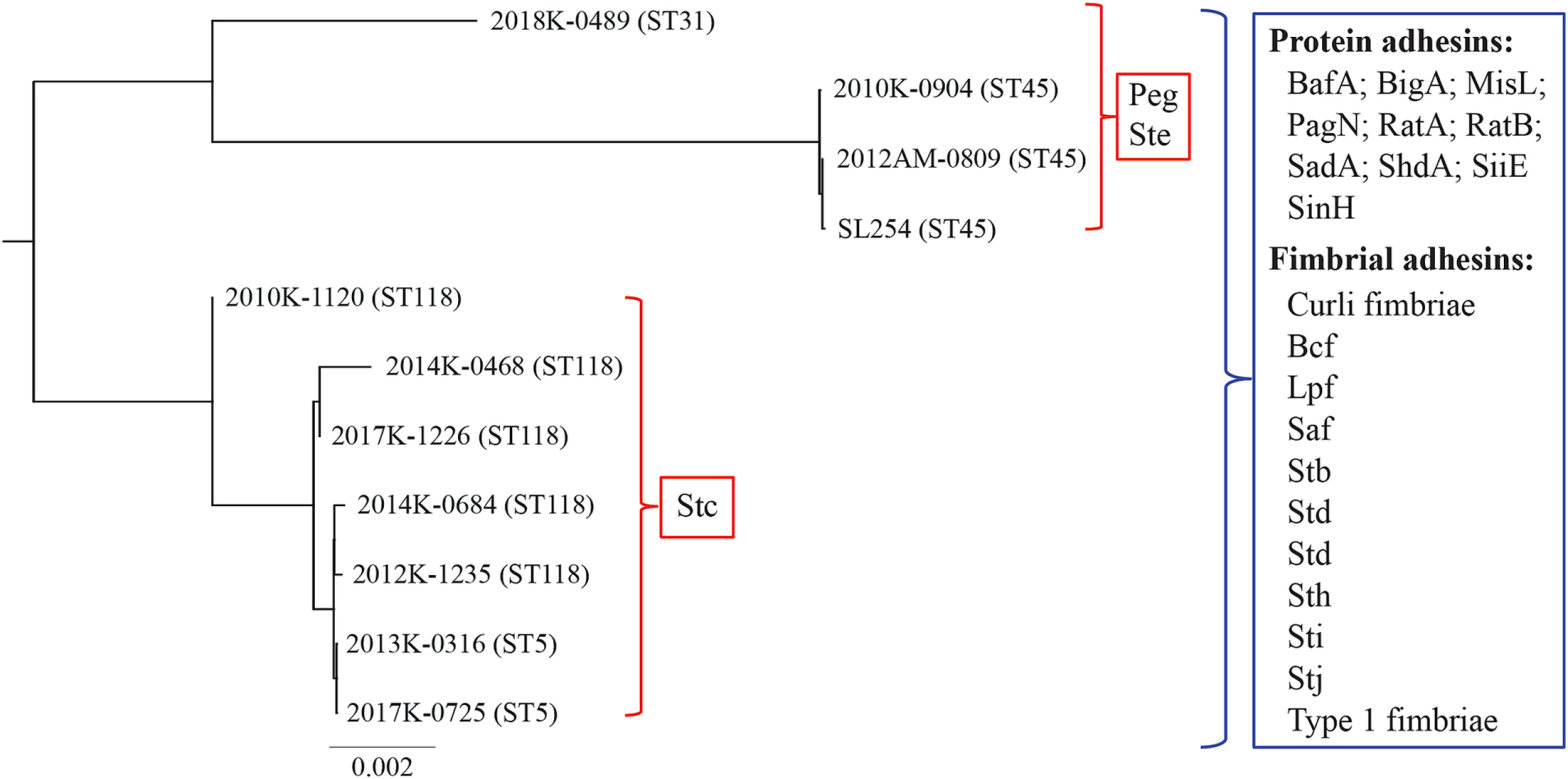
Comparative analysis of fimbrial and adhesin genes in *S. enterica* Newport strains. Genes encoding protein or fimbrial adhesins in each strain were concatenated in a conserved order. The concatenated sequences were aligned using Clustal Omega (1.2.3) in Geneious Prime® (2025.0.3) and a consensus tree was constructed using Jukes-Cantor method for genetic distance model and Neighbor-Joining method tree build. The tree was resampled using Bootstrap method with 10,000 replicates. Adherence factors listed in the blue boxes are those detected in all strains examined while adherence factors listed in the red boxes are those detected only in a subset of strains as shown in the figure.

A total of 104 genes related to the biogenesis of 20 fimbriae were examined in detail (Supplementary Table 3). Homologs of genes encoding bovine colonization factor (Bcf) (*bcfABCDEFG*), curli fimbriae (*csgGFED* and *csgBAC*), long polar fimbriae (Lpf) (*lpfABCDE*), Stb fimbriae (*stbABCDE*), Std fimbriae (*stdABC*), Stf fimbriae (*stfACDEFGH*), Sth fimbriae (*sthABCDE*), Sti fimbriae (*stiABCH*), Stj fimbriae (*stj1*, *stj2*, *stjCB*, and *stj3*), and type 1 fimbriae were detected in all Newport strains examined, while no homologs of the genes related to biogenesis of plasmid-encoded fimbriae (Pef) (*pefBACD*), *Salmonella enterica* fimbriae (*Sef*) (*sefABCD*), Sta fimbriae (*staABCDEFG*), Stg fimbriae (*stgABCD*), Stk fimbriae (*stkABCDEFG*), or Typhi colonization factor (*tcfABCD*) were identified in any of the strains examined. Furthermore, homologs of genes related to biogenesis of Peg fimbriae (*pegABCD*) and Ste fimbriae (*steABCDEF*) were identified only in the lineage II strains (ST45 and ST31) while homologs of genes related to biogenesis of Stc fimbriae (*stcABCD*) were only identified in the lineage III strains (ST118 and ST5) (Figure 4). Interestingly, genes encoding Pef fimbriae (*pegABCD*) in the lineage II strains were located at the same chromosomal location as the genes encoding Stc fimbriae (*stcABCD*) in the lineage III strains. Genes encoding Ste fimbriae (*steABCDEF*) in the lineage II strains were located between *mazG* and *relA*, and this location was occupied by a 144 bp *steA* pseudo gene in the lineage III strains, implying evolutionary elimination of *ste* gene cluster in the ST118 and ST5 strains.

### 3.6 Analyses of phenotypic traits related to attachment and biofilm formation

**Figure 5:**
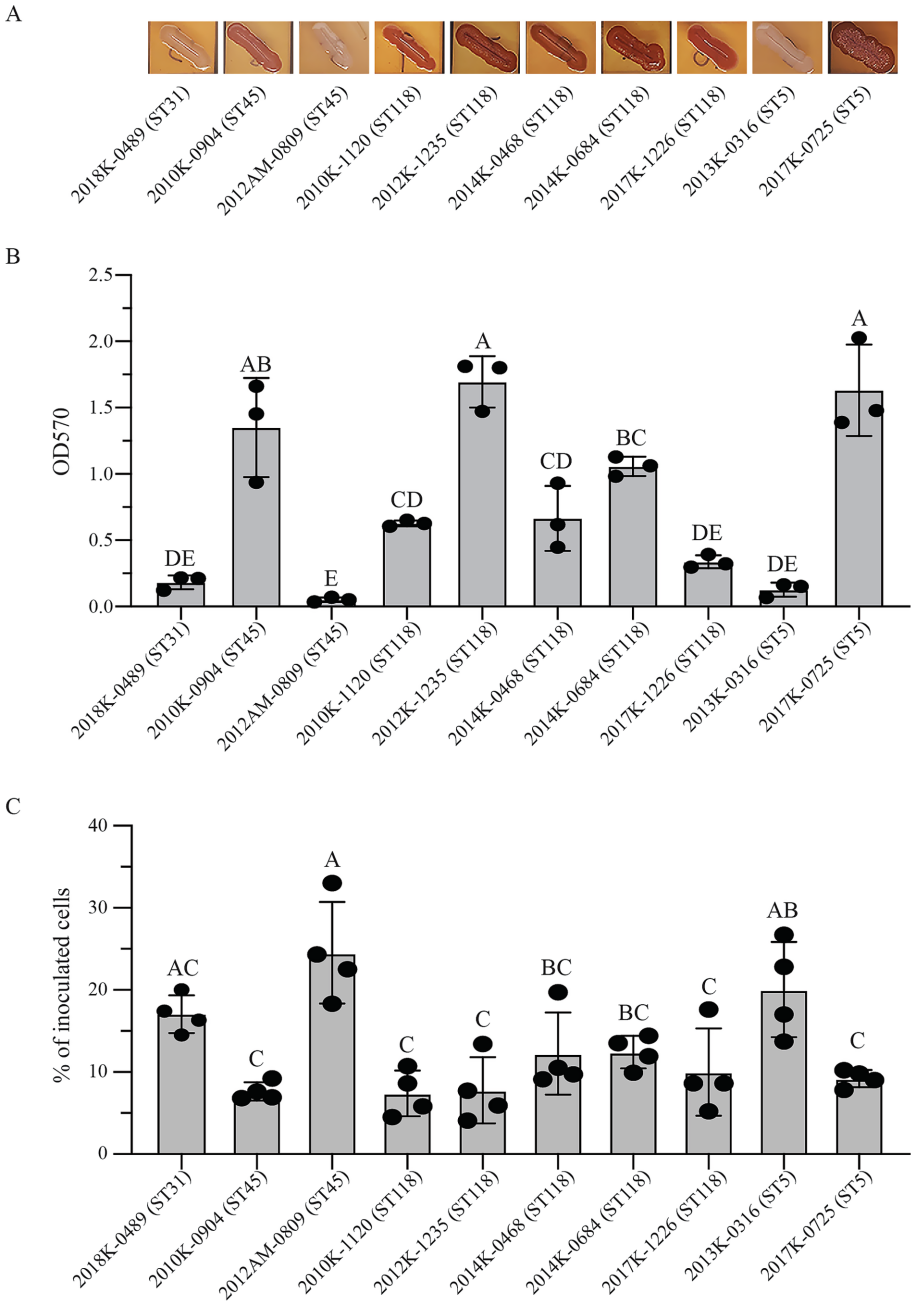
Curli production, biofilm formation, and attachment to cantaloupe. **(A)** Detection of curli fimbriae on CRI plates. Curli fimbriae were examined by growing each strain on the CRI plates at 28 °C for 48 h. Production of curli fimbriae is indicated by red colonies which resulted from the binding of CR dye supplemented in growth medium. **(B)** Quantitative analyses of biofilms under a static growth condition at 28 °C for 48 h. The attached biomass on each tube was stained by crystal violet and quantified by the absorbance at 570 nm as detailed in Material and Methods. Each data set represents the mean and SD of three biological replicates. Differences that are statistically significant (One-way ANOVA followed by a Tukey’s multiple comparisons test, adjust *p* < 0.05) are indicated by the different letters. **(C)** Quantitative analyses of attached Newport cells on the cantaloupe rind following spot inoculation as detailed in Material and Methods. Each data set is the mean and SD of four biological replicates expressed as the percent of the inoculated cells. Differences in the attachment among the strains were determined by One-way ANOVA followed by a Tukey’s multiple comparisons test, adjust *p* < 0.05. Differences that are statistically significant are indicated by different letters.

All Newport strains were motile when grown in LB media and incubated at 37 °C or 28 °C (Data not shown). Expression of curli fimbriae at 28 °C were observed for most strains but variations were observed between the strains of the same ST (Figure 5A). For example, ST45 strain 2010K-0904 produced curli fimbriae but ST45 strain 2012AM-0809 was curli-deficient. ST5 strain 2017K-0725 produced curli fimbriae but ST5 strain 2013K-0316 was curli-deficient. All five ST118 strains expressed curli fimbriae while ST31 strain 2018K-0489 was curli-deficient under the condition examined. Biofilm formation of *S. enterica* Newport on glass surfaces appeared to be strongly associated with curli fimbriae since curli-expressing strains produced significantly greater amount of surface-attached biomass than the curli-deficient strains (Unpaired t test, *p* < 0.0001). Among the curli-producing strains, strain variation in biofilm formation was also observed. ST118 strain 2012K-1235 and ST5 strain 2017K-0725 produced the greatest amount of surface-attached biomass among all strains tested and were significantly greater than any of the other strains except ST45 strain 2010K-0904 (Figure 5B).

Like biofilm formation, attachment to cantaloupe appeared to be strain dependent and unassociated with either ST or lineage. For example, a significant difference in cell population attached to the cantaloupe rind was revealed between the two ST45 strains and between the two ST5 strains (Ordinary one-way ANOVA followed by Tukey’s multiple comparisons test, adjust *p* < 0.05) (Figure 5C). However, in contrast to the biofilm formation, attachment of *S. enterica* Newport to cantaloupe rind was lessened by curli fimbriae under the condition tested since the attached Newport population on the cantaloupe for the curli-deficient strains was significantly greater than the curli-producing strains (Unpaired t test, *p* < 0.0001). For curli deficient strains, there were about 20.5% of inoculated cells retained on the cantaloupe on average after wash, while for the curli-expressing strains, only about 9.5% of inoculated cells were retained on the cantaloupe.

## 4 Discussion

*S. enterica* Newport is a polyphyletic serotype that consists of three main lineages exhibiting considerable differences in host association, antimicrobial resistance, and geographic specific traits (Sangal et al., 2010; Zheng et al., 2017; Hudson et al., 2023). This genetic diversity is thought to be contributing greatly to the ecological success of Newport considering that Newport strains can infect a wide range of hosts, colonize diverse niches, and contaminate a wide variety of food products. The Newport strains examined in our study were selected based on their epidemiological links to fresh produce or plant seeds using the data available to the CDC before 2021. More lineage III strains were revealed, and among the lineage III strains, ST118 appeared to be a main ST. However, this epidemiological association does not seem to be fresh produce/plant seeds specific, rather, it likely resulted from the overall prevalence of ST118 in the United States. This speculation is supported by the analysis of STs of other Newport genomes deposited in GenBank as of June 2025. A total of 47 SNP clusters containing more than 100 matched *S. enterica* Newport strains were retrieved from the Pathogen Detection in June 2025 (Supplementary Table 4), among which, 28 were ST118 (60%), seven were ST5 (15%), and seven were ST45 (15%), confirming the prevalence of ST118 in the United States.

Recent application of whole genome sequencing in monitoring foodborne disease outbreaks and epidemiological investigations has greatly improved the resolution of strain typing. Strains belonging to the same ST can be further classified based on the SNPs in the core genome (cgMLST). The ten Newport strains examined in our study were placed in 10 distinct SNP clusters that differed greatly in the number of matched strains. The meta data associated with the matched strains within each SNP cluster provided valuable information for understanding prevalence and distribution of various Newport genotypes. For example, among the five SNP clusters harboring the ST118 strains examined, PDS000127718 represents a persistent genotype and has been linked to several outbreaks of Newport infection since 2014. Although PDS000127718 strain was detected as early as in 1979, it remained low till 2014, when the number of PDS000127718 strains deposited to public databases started to increase greatly, which was likely due to the occurrence of the 2014 cucumber-associated outbreak in the United States (Angelo et al., 2015). Since 2014, the number of PDS000127718 strains continued to increase and reached a peak in 2022. The total number of matched strains reached to 4,994 in May 2025 (PDS000127718.405), implying continued human infections by PDS000127718 strains considering that most of strains reported in 2025 were clinical. As of June 2025, the SNP cluster PDS000127718 remained to be the largest SNP cluster containing *S. enterica* Newport strains (Supplementary Table 4). The second largest SNP cluster containing *S. enterica* Newport strains was PDS000029636, which was also identified in our study and carried ST5 strains including 2017K-0725. The earliest PDS000029636 strain was detected in 2003, and the number of the strains started to increase in 2015 and reached a peak in 2022. PDS000029636 represents another persistent genotype and strains within this cluster have been linked to several large outbreaks, including the 2023 multistate outbreak of Newport infections associated with consumption of melons in the United States (Cataldo et al., 2025).

The ability of *Salmonella* to cause diseases in humans and animals, as well as their ubiquitousness in natural environments is attributed at least in part to their genomic plasticity. Among the 17 pathogenicity islands identified in *Salmonella*, some carry essential virulence determinants while others possess genes contributing to host and/or niche adaptation. Among the seven pathogenicity islands detected in Newport strains, SPI-1, SPI-2, SPI-4, SPI5, and SPI-9 were highly conserved (>90% sequence identity) while SPI-3 and SPI-6 exhibited considerable sequence variation. The key features conferred to the Newport by the SPIs include secretion systems (T1SS, T3SS, and T6SS), metal transporters (iron, manganese, and magnesium), and fimbrial and nonfimbrial adhesins (BapA, MisL, SiiE, and Saf fimbriae).

The SPI-1 encoded T3SS is required for intestinal epithelial invasion through direct translocation of effector proteins from bacterial cytoplasm to the host cells. Interestingly, the majority of Newport strains (10 out of 11 strains examined) had a mutation in the gene encoding the type III secretion system YopJ family effector AvrA. The mutations included a nucleotide insertion in the *avrA* of ST45 and ST5 strains, and an insertion, a deletion, or a transversion in the *avrA* of ST118 strains. All mutations led to a premature stop codon, implying a natural silence of AvrA function in the Newport strains. AvrA was reported to negatively influence the host response triggered by *Salmonella* to limit host cellular damages (Du and Galan, 2009). Our data support the speculation that AvrA effector is likely under a strong evolutionary pressure for altered function, like in a previous study on an allelic variant of AvrA with impaired activity in *S. typhimurium* strain SL1344 (Du and Galan, 2009). A mutation was also identified in the T3SS effector gene *sopA*, located outside of SPI-1. Nearly half of the Newport strains had a mutation in *sopA*, including an insertion in the gene of both ST5 strains, and a deletion, an insertion, and two transitions in the gene of four ST118 strains (Supplementary Table 2). SopA was suggested to modulate host inflammatory responses by directly targeting innate immune signaling (Kamanova et al., 2016). The high frequency mutation in *sopA* may suggest that, like *avrA*, *sopA* is under a selection pressure.

Unlike the SPI-1 encoded T3SS, no mutations were identified in any of the T3SS genes located on the SPI-2. The SPI-2 encoded T3SS translocates effector proteins across the *Salmonella*-containing vacuole membrane into the macrophage cytosol, thus is required for pathogen survival in macrophages (Kuhle and Hensel, 2004). Among the known effector proteins translocated by the SPI-2 encoded T3SS, three are located on the SPI-2 (*ssaB*, *sseF*, and *sseG*). All three retained a wild type in the Newport strains examined. Among the effector genes located outside of SPI-2, homologs of nine were identified in all Newport strains examined while homologs of four genes (*gogB*, *sseI*, *sseK1* and *sspH1*) were detected only in a subset of strains. No association was identified between the lineage or ST and the T3SS effector genes repertoire.

T1SS is common in pathogenic bacteria, and secretes toxins, adhesins, iron-scavenger proteins, lipases, or proteases in one step across two membranes into the extracellular environment (Spitz et al., 2019). The secreted proteins may be released, remain on the surface of bacterial cell, or be injected into the target cell (Costa et al., 2015). The SPI-4 carries genes (*siiABCDEF*) that are required for the translocation of the large protein adhesin SiiE to the bacterial cell surface. While SiiF (an ABC transporter), SiiD (a periplasmic adaptor protein), and SiiC (an outer membrane protein) are components of T1SS, SiiA and SiiB function as accessory proteins to retain the secreted SiiE on the bacterial cell surface, and SiiE contributes to the invasion of polarized epithelial cells (Barlag and Hensel, 2015). Like SPI-4, the SPI-9 carries four genes (*bapABCD*) encoding the large protein adhesin BapA and the T1SS (BapBCD) for translocation of BapA. The secreted BapA appears to be loosely attached to the bacterial cell surface, contributing to biofilm formation and host colonization in *S. enterica* serotype Enteritidis (Latasa et al., 2005). Conservation of SPI-4 and SPI-9 implies a role of SiiE and BapA in pathogenesis, nonhost survival, and niche adaption in *S. enterica* Newport.

T6SS translocates effector proteins directly into prokaryotic and eukaryotic cells and contributes to pathogenesis and interbacterial competition (Navarro-Garcia et al., 2019; Wood et al., 2020). In *S. enterica*, five T6SS gene clusters have been reported, located on SPI-6, SPI-19, SPI-20, SPI-21, and SPI-22, respectively (Blondel et al., 2009). Unlike T3SS, T6SS is not conserved in *S. enterica*. Genomic content of T6SS genes as well the T6SS encoded function appears to be serotype and even strain dependent. In serotype Dublin, both SPI-6 and SPI-19 encoded T6SS contributed to bacterial virulence, host colonization, and interbacterial competition (Amaya et al., 2022). T6SS contributes to pathogenesis in Typhimurium but played a minimal role in Gallinarum (Mulder et al., 2012; Schroll et al., 2019). Our study identified a SPI-6 encoded T6SS in all Newport strains examined. Genes encoding the 13 core components of T6SS were present in most Newport strains examined. In two ST118 strains, a large deletion (~24 Kb) spanning from *tssK* (gene position: 352–1,344) to *safC* (gene position: 1–744) was identified. This deletion eliminated genes encoding key components of T6SS membrane complex, TssL and TssM, thus impairing T6SS in both strains, and abolished Saf fimbriae simultaneously. Additional studies are needed to understand the physiological and pathogenic impact conferred by this large deletion.

Unlike the SPIs, lineage-specific fimbriae were identified in *S. enterica* Newport. All lineage II strains (ST45 and ST31) carried Peg and Ste fimbriae genes but lacked Stc genes. In contrast, all lineage III strains (ST118 and ST5) carried Stc fimbriae genes but lacked Peg and Ste genes. More than 30 different types of fimbriae were reported for *S. enterica* (Yue et al., 2012). In this study, a core set of fimbriae was revealed in Newport, including Bcf, curli fimbriae, Lpf, Saf, Stb, Std, Stf, Sth, Sti, Stj, and type I fimbriae. In Typhimurium, Lpf, Bcf, Stb, Stc, Std, and Sth fimbriae appeared to be important for the intestinal persistence of pathogen in mice, while curli, type 1 fimbriae, Pef, or Stf fibarie had a minimal impact (Weening et al., 2005). In Enteritidis, elimination of Stb or Peg fimbriae resulted in an impaired colonization of pathogen in chick caeca while deficiency in Bcf, curli, Lpf, Saf, Sef, Std, Ste, Stf, Sth, Sti, or type 1 fimbriae did not impact the colonization of the pathogen in chicken intestines significantly (Clayton et al., 2008). Therefore, function of a particular fimbriae or a set of fimbriae in *S. enterica* appears to be serotype or even strain dependent. Additionally, fimbrial genes are often under strong evolutionary selection, especially for the genes encoding adhesins, which are located at the tip of the mature fimbriae mediating specifically binding between the pathogen cell and host receptors. In Newport, allelic variations are present in multiple adhesin genes including *fimH*, *bcfD*, and *stfH*, and contribute to the host tropisms (De Masi et al., 2017). Our study suggested congruent evolution of FimH, BcfD, and StfH, like several SPIs detailed in this study, supporting lineage-specific divergence in Newport population, an evolution trend also observed in serotype Montevideo (Nguyen et al., 2018).

Although genes encoding curli fimbriae were present in all Newport strains examined, expression of curli fimbriae was not detected in every strain. Failure to express curli fimbriae in the ST5 strain 2013K-0316, ST31 strain 2018K-0489, and ST45 strain 2012AM-0809 could not be explained by a loss-of-function mutation in any of the curli genes, or in the genes encoding transcriptional regulators of curli genes, such as *rpoS* or *rcsB* as we reported previously in *Escherichia coli* O157: H7 (Carter et al., 2012; Carter et al., 2014). Curli enhanced biofilm formation of *E. coli* O157: H7 on stainless steel and glass surfaces and contributed to the development of mixed biofilms by *E. coli* O157: H7 and spinach leaf-associated microorganisms (Carter et al., 2016; Carter et al., 2019). Curli were implicated in the biofilm formation of *S. enterica* Typhimurium on abiotic surfaces but not required for attachment and biofilm development of *S. enterica* Typhimurium on the hyphae of *Aspergillus niger* (Brandl et al., 2011; Trmcic et al., 2018). Our study supports a role of curli in nonhost survival and persistence in *S. enterica* Newport, similarly to a role of curli fimbriae in *S. enterica* Enteritidis (Austin et al., 1998), considering the increased biofilms on glass surfaces for the curli-expressing Newport strains when compared with the curli-deficient Newport strains. Contribution of curli fimbriae to the initial attachment and colonization of enteric pathogens on fruits and vegetables varies and often depends on the experimental systems. Curli promoted the attachment of *E. coli* O157: H7 to lettuce leaves when pathogen cells were spot inoculated on the leaves (Fink et al., 2012) but were not required for attachment of *E. coli* O157: H7 to sprouts (Torres et al., 2005). Curli enhanced the attachment of Newport cells to alfalfa sprouts and cucumbers (Barak et al., 2005; Jung and Schaffner, 2022) but had a minimal role in attachment of Typhimurium to parsley leaves (Lapidot et al., 2006). Our study revealed a modulating effect of curli in the attachment of Newport to cantaloupe rind when pathogen cells were spot inoculated, affirming the complex interactions between human pathogen cells and plant tissues. Improved adherence of curli-deficient Newport on cantaloupe rind suggests presence of other adhesins expressed in the curli-deficient strains, which may mediate adherence of pathogen cells to cantaloupe rind. Comparative transcriptomic studies would provide insights into the molecular basis of the phenotypic traits observed here.

Our study uncovered genomic traits in a set of Newport strains linked to fresh produce-associated outbreaks or sporadic infections during 2010–2018. ST118 appeared to be a main sequence type associated with fresh produce or seed products and also a dominant ST in the United States. Identification of persistent SNP clusters provided genomic markers for high-risk Newport strains, which have potential to persist in environments and to cause large outbreaks. Although the core virulence determinants were conserved in the Newport strains, variations in the virulence genes repertoire were detected, which were mainly introduced by the loss-of-function mutations and gene duplications in genes encoding T3SS effectors, T6SS, and fimbrial and protein adhesins. Swimming motility was detected in all Newport strains, however, expression of curli fimbriae was not observed in every strain. Curli fimbriae enhanced biofilm formation on glass surfaces but tempered attachment of Newport cells to cantaloupe rind, implying a role of other adhesins in the initial interaction between human pathogen cells and the surface of cantaloupe rind. *S. enterica* Newport harbor’s many fimbrial and protein adhesins. Understanding conditions and ecological niches that promote the expression of these adhesins would provide fundamental knowledge about host range, ecological niches, as well as the linkages of *S. enterica* Newport with certain food products including fresh vegetables and fruits.

## Data Availability

The datasets presented in this study can be found in online repositories. The names of the repository/repositories and accession number(s) can be found in the article/Table 1.

## Author disclaimer

Mention of trade names or commercial products in this publication is solely for the purpose of providing specific information and does not imply recommendation or endorsement by the U.S. Department of Agriculture. USDA is an equal opportunity provider and employer. The findings and conclusions in this report are those of the authors and do not reflect the view of the Centers for Disease Control and Prevention, the Department of Health and Human Services, or the United States government. Furthermore, the use of any product names, trade names, images, or commercial sources is for identification purposes only, and does not imply endorsement or government sanction by the U.S. Department of Health and Human Services.
